# Assessment of dental students’ knowledge and performance of master gutta-percha cone selection and fitting during root canal treatment: a pilot study

**DOI:** 10.1186/s12909-024-05347-9

**Published:** 2024-04-04

**Authors:** Sarah M. Alkahtany, Shaima E. Alabdulkareem, Wajd H. Alharbi, Norah F. Alrebdi, Tomather Sultan Askar, Sundus M. Bukhary, Amal A. Almohaimede, Kholod Khalil Al-Manei

**Affiliations:** 1 Department of Restorative Dental Sciences, Division of Endodontics, College of Dentistry, King Saud University, 11527 Riyadh, PO Box 68004, Saudi Arabia; 2 General Dentist, College of Dentistry, King Saud University, 11527 Riyadh, PO Box 68004, Saudi Arabia

**Keywords:** Master cone, Gutta-percha, Endodontics, Obturation, Dental education, Dental student

## Abstract

**Background:**

Misconceptions should be detected early in dental students’ training to improve their clinical performance. Accordingly, this study aimed to assess undergraduate dental students’ knowledge and performance of master gutta-percha (GP) cone selection and fitting during clinical endodontic courses at the College of Dentistry, King Saud University.

**Methods:**

Ninety-nine undergraduate dental students completed an online survey about their knowledge of master GP cone selection. Forty-five of these students were observed by faculty members in clinical endodontic courses while they fitted master GP cones during root canal treatments. The observers recorded the details of each student’s cone-fitting techniques. The data were analysed using t-tests, one-way analysis of variance, and chi-square tests (*p* < 0.05). Inter- and intra-observer reliability were tested using Fliess’ Kappa.

**Results Survey:**

All participants had good knowledge of over-extended cone management, while 80.8% knew how to properly manage a short cone. The proper flaring assessment method was selected by 86.9% of the female and 34.2% of the male students, and this difference was statistically significant (*p* = 0.0001).

**Observation:**

The students labelled the working length on the master GP cone with an indentation in 64.4% of the cases and by bending the cone 35.6% of the time. Of all students, 84.4% encountered an apical stop, and this rate correlated significantly with the length of the cone on the master apical cone radiograph (*p* = 0.001). Improper shaping of the canal was the most common cause of ill-fitting cones (83.3%), while 16.7% of the students chose the wrong cone size. The final obturation length was adequate in 80% of the cases; 57.8% of the students were helped by instructors.

**Conclusions:**

Most students had the basic knowledge required to solve problems related to the selection of master GP cones. However, in the clinical setting, more than half of the students required the assistance of an instructor to adjust their cone’s fit. The presence of an apical stop had the most significant effect on the length of the fitted master GP cone on radiography. The most common cause of ill-fitting master cones was improper shaping of the canal.

## Introduction

Root canal obturation is a part of root canal treatment (RCT) that is necessary to prevent leakage, inhibit bacterial contamination, and create a healthy environment for periapical tissues to heal [1, 2]. Gutta-percha (GP) is the most widely used core-filling material. It is usually combined with root canal sealers to obturate the root canal space [3]. Obturation can be performed using many techniques, including cold lateral and warm vertical condensation. Cold lateral condensation remains the most commonly used method for obturation because of its reasonable cost, relative technical ease, superior length control, and minimal sealer extrusion compared to other techniques [4, 5].

Procedural accidents are undesired circumstances that can occur during any step of RCT and affect treatment outcomes. Obturation-related procedural accidents, such as underfilling or overfilling the root canal, are commonly reported at undergraduate clinics [6]. Overfilling can occur in immature root apexes or in cases in which apical constriction has been lost due to pathological root resorption. Moreover, cleaning and shaping can extend beyond the apical foramen due to inaccurate working-length determinations, resulting in over-instrumentation, which leads to filling material being extruded out of the root canal system [7–10]. The success and healing rate of RCT was found to be lower in cases of overfilling [11]. Root canal filling extrusion has also been correlated with delayed healing in periapical lesions [12].

Overfilling can be prevented by the clinician with proper apical preparation, adequate flaring, and accurate master GP cone selection and fitting. According to the treatment standards of the American Association of Endodontics (2020), the GP cone should be adjusted to fit the length, apical size, and taper of the prepared root canal. A well-selected master cone should create a tug-back sensation, which indicates resistance to displacement. Just before obturation, a master apical cone (MAC) radiograph should be taken to confirm the length of the selected cone [3, 13].

Undergraduate dental students acquire the knowledge and skills required to perform endodontic treatment during their training. Different students demonstrate different learning capabilities and develop proficiency at different speeds [14–16]. Moreover, in addition to misconceptions that arise from an insufficient understanding of principles, a lack of experience might affect students’ future clinical performance and lead to procedural accidents, including obturation-related mishaps [17]. These misconceptions should be detected early in training to improve the outcomes of endodontic treatments performed by students, and teaching should be improved for each step of the treatment, including obturation and master GP cone selection and fitting [18].

Undergraduate endodontic education at our institute (College of Dentistry, King Saud University) includes two courses: preclinical (in the third academic year) and clinical endodontics (in the fourth academic year). The pre-clinical endodontic curriculum is the cornerstone that teaches students basic clinical and biological principles. It offers hands-on training in the fundamental technical procedures of non-surgical RCT, including tooth isolation, access cavity preparation, cleaning and shaping with both hand and rotary systems, lateral compaction obturation techniques, and tooth temporisation on extracted natural teeth and plastic models of teeth.

The students learn to perform a root canal obturation using cold lateral compaction technique with 0.02-taper GP cones. Proper master GP cone selection involves fitting a cone that is the same size and length as the master apical file. The cone is marked to indicate the working length at the same reference point used during canal shaping. The master GP cone should resist displacement in the coronal direction (tug-back) and extrusion out of the canal in the apical direction (apical stops). In addition, the root canal flaring should be evaluated before proceeding with the obturation by inserting an endodontic spreader with a master GP cone 1–2 mm shorter than the working length (WL). A MAC radiograph should then be taken to verify the length of the fitted cone and determine whether it is adequate (0–2 mm from the apex), short (> 2 mm shorter than the root apex), or over-extended (it extends beyond the root apex). If the cone length on the radiograph is short or over-extended, the student is instructed to adjust the cone or re-instrument the canal to correct the ill-fitting cone.

On the other hand, the clinical endodontic course at our institute builds on the knowledge, skills, and practice obtained in the preclinical course to teach students how to perform non-surgical RCT. The students execute the RCT independently, receiving verbal feedback and approval after each step (access cavity formation, working length determination, MAC radiography, and final radiography). Students may face challenges in adapting to the sudden transition from preclinical training to the clinical situation. When a student encounters difficulties or mishaps, the instructor may provide manual assistance to help the student overcome the challenge or remedy the mishap [19].

No extant research was found regarding dental students’ knowledge and performance of master GP cone selection and fitting during RCT. Therefore, our study was designed to assess undergraduate dental students’ knowledge of master GP cone selection and closely observe and evaluate the clinical skills and techniques used to fit a master GP cone during clinical endodontic courses at the College of Dentistry, King Saud University.

## Materials and methods

The study protocol was approved by the university’s institutional review board (Approval No E-22-7091). Consent forms were signed by the participants to authorise the survey and clinical observation. Fourth- and fifth-year dental students participated in this study during the 2022–2023 academic year.

### Sample size calculation

We calculated the required sample size using G*Power software (version 3.1.9.4). For the knowledge survey with an alpha of 0.05, an estimated effect size of 0.38, and a power of 0.9, the minimum sample size should be 92 students. For the clinical skills observation with an alpha of 0.05, an estimated effect size of 0.5, and a power of 0.85, the minimum sample size should be 43 students.

### Phase I: knowledge survey

A cross-sectional survey was constructed online using Google Forms (Google Inc., US). The survey questions were designed to address clinical problems related to master GP cone selection and fitting. The options provided for each question were based on the performance of students from previous years in addition to the basic knowledge they were taught in class. A professor with relevant expertise reviewed the survey questions, and the survey was then pretested in a pilot study with 44 dental interns who had just completed their undergraduate clinical training. The questions were refined based on their responses and feedback.

Finally, a link to the survey was sent to all 160 dental students in their fourth and fifth years at the College of Dentistry, King Saud University. The survey was composed of 10 questions:

- Questions 1 and 2 sought demographic data.
- Question 3 asked about reference points.
- Question 4 enquired about canal flaring assessment.
- Question 5 asked about GP cone labelling methods.
- Questions 6 to 10 enquired about the management of various clinical problems that could emerge during the GP cone selection process.

### Phase II: clinical skills observation

For the clinical observation phase of our cross-sectional study, we recruited 45 fourth- and fifth-year female undergraduate dental students attending the College of Dentistry at King Saud University. All fourth-year students who are participating in the study have successfully fulfilled the preclinical endodontic course’s prerequisites by completing an average of 10 RCTs. Similarly, fifth-year students have fulfilled the prerequisites of both the preclinical and clinical endodontic courses by completing an average of 18 RCTs. The students were observed during their clinical endodontic courses while performing non-surgical RCTs. Only cases with a single canal were included for standardisation purposes. Cases involving calcification, open apexes, apical perforation, or root resorption were excluded.

Every student completed the cleaning and shaping before proceeding to fit the master GP cone, which had a size comparable to the master apical file (MAF) size (with a minimum size of 30). Every student evaluated the cone fitting by considering the length, apical stop, and tug-back. If the cones do not fit properly, the instructor identifies whether the problem is due to incorrect shaping or selecting the wrong cones. The student can then take appropriate action to address the issue. When the cones had been extended beyond the WL, the student either readjusted them or substituted larger cones. Re-instrumentation was necessary in cases where a short cone was encountered. If the student is unable to clearly understand the verbal instructions, the instructor will provide physical assistance to make the necessary adjustments and guide the student as they observe the technique in order to acquire the skill of cone fitting.

All students performed an obturation with cold lateral condensation using 0.02-taper GP cones (SureDent Co., Gyeonggi-Do, Korea), AH Plus sealer (Dentsply Maillefer, Ballaigues, Switzerland), and the NiTi finger spreader (Dentsply Maillefer, Ballaigues, Switzerland). The relevant endodontic procedural terminology is defined in Table 1.

**Table 1:** Definition of procedural endodontic terminology used in our study

Term	Definition
Master GP cone	The largest gutta-percha cone that can be placed either to full working length or to within a short distance of working length (0.5 mm or less), of the completely prepared root canal prior to obturation [20].
Tug-back	Slight frictional resistance of a master point to withdrawal when seated; indicates a relative degree of adaptation, at least in two dimensions [20]. • Assessed by pulling the cone and feeling resistance to the displacement in the coronal direction.
Apical stop	The matrix of dentin or other materials at the apical end of a root canal preparation that prevents further advancement or progression of both endodontic instruments and obturation materials [20]. • Assessed by pushing the cone and feeling resistance to the extrusion in the apical direction.
Reference point	The point on the occlusal or incisal tooth surface from which measurements are made
Ill-fitting cone	Either short (more than 2 mm from the apical foramen) or over-extended (extends beyond the apical foramen)
Master apical cone radiograph	Radiograph taken to confirm the length of the selected cone
Final radiograph	Radiograph taken postoperatively to evaluate the quality of the obturation

The clinical observations were conducted by four evaluators who are certified endodontists and assistant professors with a minimum of 10 years of educational experience. They were the same instructors assigned to the clinical endodontic courses with a 1:5 instructor-to-student ratio. The evaluators closely observed each student during cone selection without intervening. The following data were collected for each student during the clinical observation phase:

- Level (4th/5th year).
- Tooth type (anterior/premolar).
- Instrumentation technique (hand/rotary).
  - Hand instrumentation with the step-back technique using 0.02-taper K-files (Dentsply Maillefer, Ballaigues, Switzerland).
  - NiTi Profile rotary systems with 0.04 and 0.06 taper (Dentsply Maillefer, Ballaigues, Switzerland).
- Reference point (tooth structure/restoration).
- GP cone labelling technique (indentation/bending).
- Flaring assessment performed (yes/no).
- Presence of tug-back (yes/no).
- Presence of apical stop (yes/no).
- Number of MAC radiographs taken (1/2/3/4).
- Master GP cone length (adequate/overextended/ or short) in the MAC radiograph. The observer can then give the student instructions on how to adjust the canal preparation or the cone, as needed.
- Cause of an ill-fitting cone (improper shaping/improper cone selection).
- Method used for adjustment (if required: change the cone/cut the cone/re-instrumentation/none).
- Instructor helped the student in cone selection (yes/no).
- Obturation length in the final radiograph (adequate/ over-extended /short).

Finally, the intra- and inter-observer reliability were tested by comparing the observers’ radiographic interpretations of the master GP cone lengths with the final obturation length of 10 cases.

### Statistical analysis

Data from the survey and clinical observations were formatted as percentages and then analysed using t-tests. One-way analysis of variance (ANOVA) and chi-square tests were used to study the associations between different variables using CIBM-SPSS 26. The *p*-value was set as < 0.05. Fleiss’ kappa and interaclass correlation statistics were used to test the intra- and inter-observer reliability of different observers.

## Results

### Phase I: knowledge survey results

#### Questions 1 and 2

Out of the 160 undergraduate dental students contacted, 99 participated in the survey, yielding a response rate of 62%. Exactly 38.4% of the respondents were male, and 61.6% were female. There were 60.6% participants in their fifth year and 39.4% in their fourth year.

#### Questions 3

Most students (93.9%) selected a sound tooth structure as the most reliable reference point.

#### Question 4

**Fig. 1:**
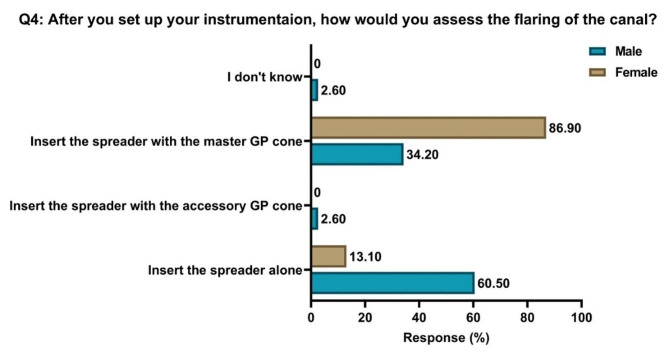
Responses in percentages of students of both genders to Question 4 concerning canal flaring assessment methods. **GP**: gutta-percha

The proper flaring assessment method was selected by 86.9% of the female and 34.2% of the male students (Fig. 1), and this difference was statistically significant (*p* = 0.0001).

#### Question 5

**Fig. 2:**
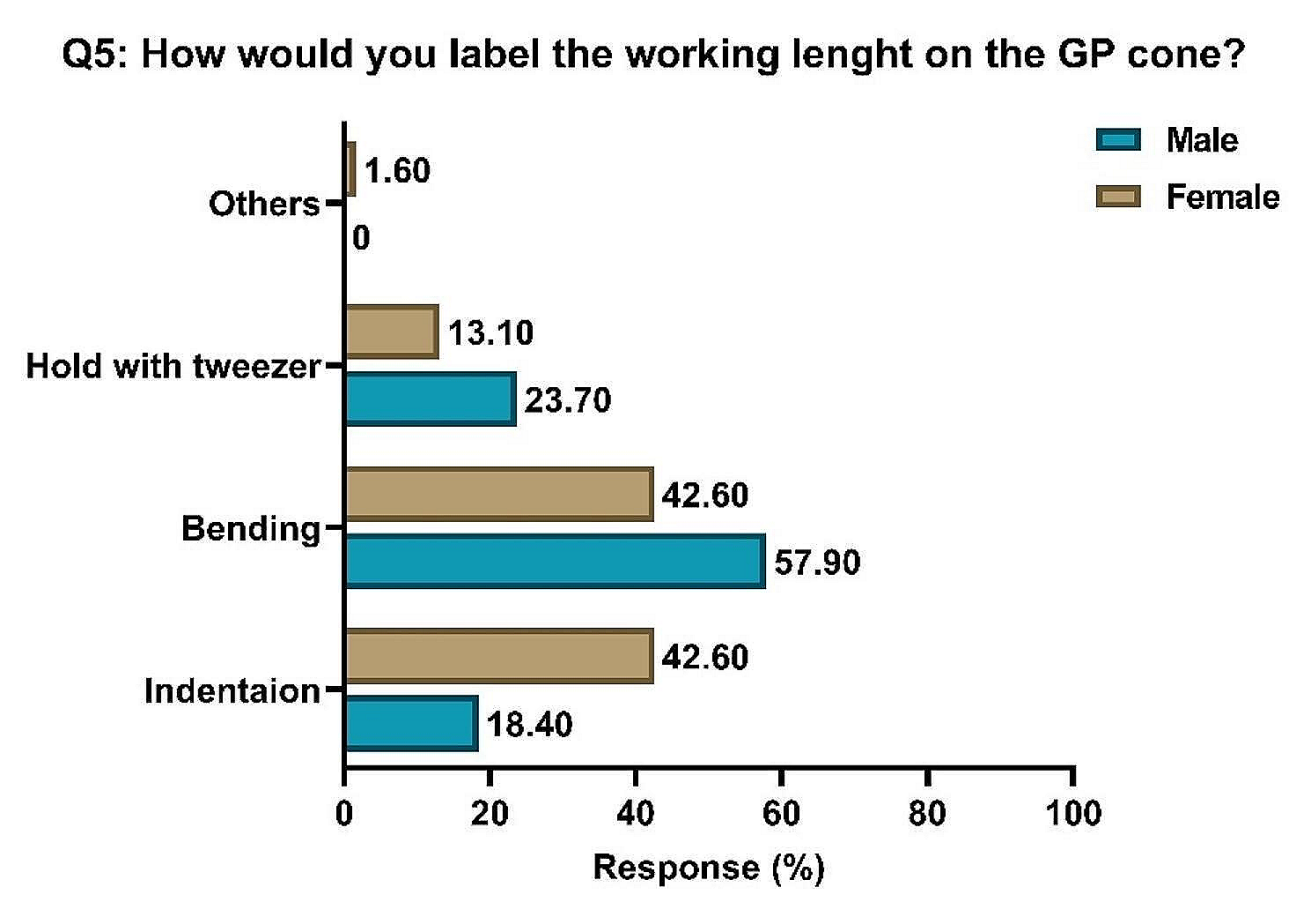
Responses in percentages of students of both genders to Question 5 concerning the best method of labelling the working length on the GP cone. **GP**: gutta-percha

Most students (48.5%) preferred to bend the cone to mark the working length, while 33.3% preferred to indent the cone (Fig. 2).

#### Questions 6 to 8

Most students (86.9%) were able to define an apical stop and tug-back. However, only 70.7% knew that the proper method of assessing an apical stop is to push the cone apically, and others selected the improper management method of pulling the cone coronally (17.2%). A few students thought that placing a cotton pellet alongside the GP cone would prevent cone movement apically (5.1%) or coronally (7.1%). Of the students, 70.7% knew how to properly manage insufficient tug-back while fitting the MAC.

#### Questions 9 and 10

All participants had a good understanding of how to manage an overextended master GP cone; 80.8% preferred to cut the tip of the cone, and 19.2% chose to re-instrument the canal. Additionally, 80.8% of the students knew that short GP cones are properly managed by checking the length of the root canal instrumentation; 15.2% opted to use a smaller cone so it could reach the full WL; and 4% preferred to proceed with the obturation using a short length.

The students’ responses are summarised in Table 2.

**Table 2:** Questions on the online survey and student responses frequency and percentages

Q. No.	Question	Responses	Frequency (%)
1	Gender	Male	38 (38.4%)
		Female	61 (61.6%)
2	Educational level	4th year	39 (39.4%)
		5th year	60 (60.6%)
3	Which of the following is the most reliable reference point during RCT?	GIC buildup	4 (4%)
		Adjacent tooth	2 (2%)
		Sound tooth structure	93 (93.9%)
4	After you set up your instrumentation, how would you assess the flaring of the canal?	Insert only a spreader 1 mm shorter than the working length (WL)	31 (31.3%)
		Insert a spreader with a master GP cone 1 mm shorter than the WL	66 (66.7%)
		Insert a spreader with an accessory cone 1 mm shorter than the WL	1 (1%)
		I don’t know	1 (1%)
5	How would you label the working length on the gutta-percha (GP)/master apical cone (MAC)?	Indentation on the GP at the determined WL	33 (33.3%)
		Bending of the GP at the determined WL	48 (48.5%)
		I just hold the GP with tweezers at the WL	17 (17.2%)
		Other	1 (1%)
6	During the MAC selection, you feel resistance when you try pushing the cone apically, but you can pull the cone without any resistance. What do you feel at this point?	There is an apical stop but no tug-back	86 (86.9%)
		There is tug-back but no apical stop	6 (6.1%)
		There is tug-back and an apical stop	6 (6.1%)
		There is no tug-back or apical stop	1 (1%)
7	While fitting the GP cone (MAC), you insert the cone to the full working length. What would you do next?	I place a cotton pellet so the GP cone will not move apically	5 (5.1%)
		I place a cotton pellet so the GP cone will not move coronally	7 (7.1%)
		I push the GP cone apically to check the apical stop	70 (70.7%)
		I pull the GP cone coronally to check the apical stop	17 (17.2%)
8	During the fitting of the GP cone (MAC), the cone reaches the full WL, but you do not feel tug-back. What should you do?	Cut the tip of the cone or use a larger cone that reaches the WL with tug-back	70 (70.7%)
		Re-instrument the canal and create an apical seat to feel the tug-back	13 (13.1%)
		Obturate the canal; no need to feel the tug-back	13 (13.1%)
		I don’t know	3 (3%)
9	During the fitting of the GP cone (MAC), the cone is over-extended, and you do not feel an apical stop. What should you do?	Cut the tip of the cone or use a larger cone that reaches the WL with an apical stop	80 (80.8%)
		Re-instrument the canal and create an apical seat to establish an apical stop	19 (19.2%)
		Obturate the canal; no need to feel the apical stop	0
		I don’t know	0
10	During the fitting of the GP cone (MAC), the cone is shorter than the WL, but you feel tug-back. What should you do? There is an apical stop but no tug-back	I will check whether the master apical file can reach the full WL	80 (80.8%)
		I will use a smaller cone so it can reach the full WL	15 (15.2%)
		I will obturate the canal; no need to increase the length	4 (4%)
		I don’t know	0

Abbreviations: **GIC**: Glass ionomer cement, **GP**: Gutta-percha, **WL**: Working length, **MAC**: Master apical cone

### Phase II: clinical skills observation results

A total of 45 female undergraduate dental students were included in the clinical observation of cone-fitting techniques, with 44% of the participants in their fourth year (*n* = 20) and 55% in their fifth year (*n* = 25). The data collected from the students are summarised in Table 3.

**Table 3:** Summary of clinical observations of undergraduate dental students performing root canal treatments

Data collected	Category	Frequency (%)
Educational level	4th year	20 (44.4%)
	5th year	25 (55.6%)
Tooth type	Anterior	22 (48.9%)
	Premolar	23 (51.1%)
Instrumentation technique	Hand	22 (48.9%)
	Rotary	23 (51.1%)
Reference point	Tooth structure	42 (93.3%)
	Restoration	3 (6.7%)
Cone labelling	Bending	16 (35.6%)
	Indentation	29 (64.4%)
Presence of tug-back	Yes	25 (55.6%)
	No	20 (44.4%)
Presence of apical stop	Yes	38 (84.4%)
	No	7 (15.6%)
Flaring assessment performed	Yes	33 (73.3%)
	No	12 (26.7%)
Number of MAC radiographs	1	20 (44.4%)
	2	19 (42.2%)
	3	5 (11.1%)
	4	1 (2.2%)
Master GP cone length in MAC radiograph	Adequate	16 (35.6%)
	Over-extended	13 (28.9%)
	Short	16 (35.6%)
Cause of ill-fitting cone	Improper shaping	25 (55.6%)
	Improper cone selection	5 (11.1%)
Method of adjustment	Change the cone	9 (20.0%)
	Cut the cone	7 (15.6%)
	Re-instrumentation	15 (33.3%)
	None	14 (31.1%)
An instructor helped the student	Yes	26 (57.8%)
	No	19 (42.2%)
Final obturation length	Adequate	36 (80.0%)
	Over-extended	3 (6.7%)
	Short	5 (11.1%)

Abbreviations: **MAC**: Master apical cone

The study included students who performed RCTs on single canal teeth: 48.9% (*n* = 22) of the treatments were completed on anterior teeth, and 51.1% (*n* = 23) on premolars. About 49% (*n* = 22) of the students used hand instrumentation, while 51% (*n* = 23) used rotary instrumentation. Most students (93.3%, *n* = 42) used a reliable reference point on the tooth structure. Exactly 35.6% (*n* = 16) of the participants bent the GP, and 64.4% (*n* = 29) made an indentation on the GP cone to label the WL. No significant correlation was found between the GP labelling technique used and the length of the master GP cone on the MAC radiograph (*p* = 0.703).

**Fig. 3:**
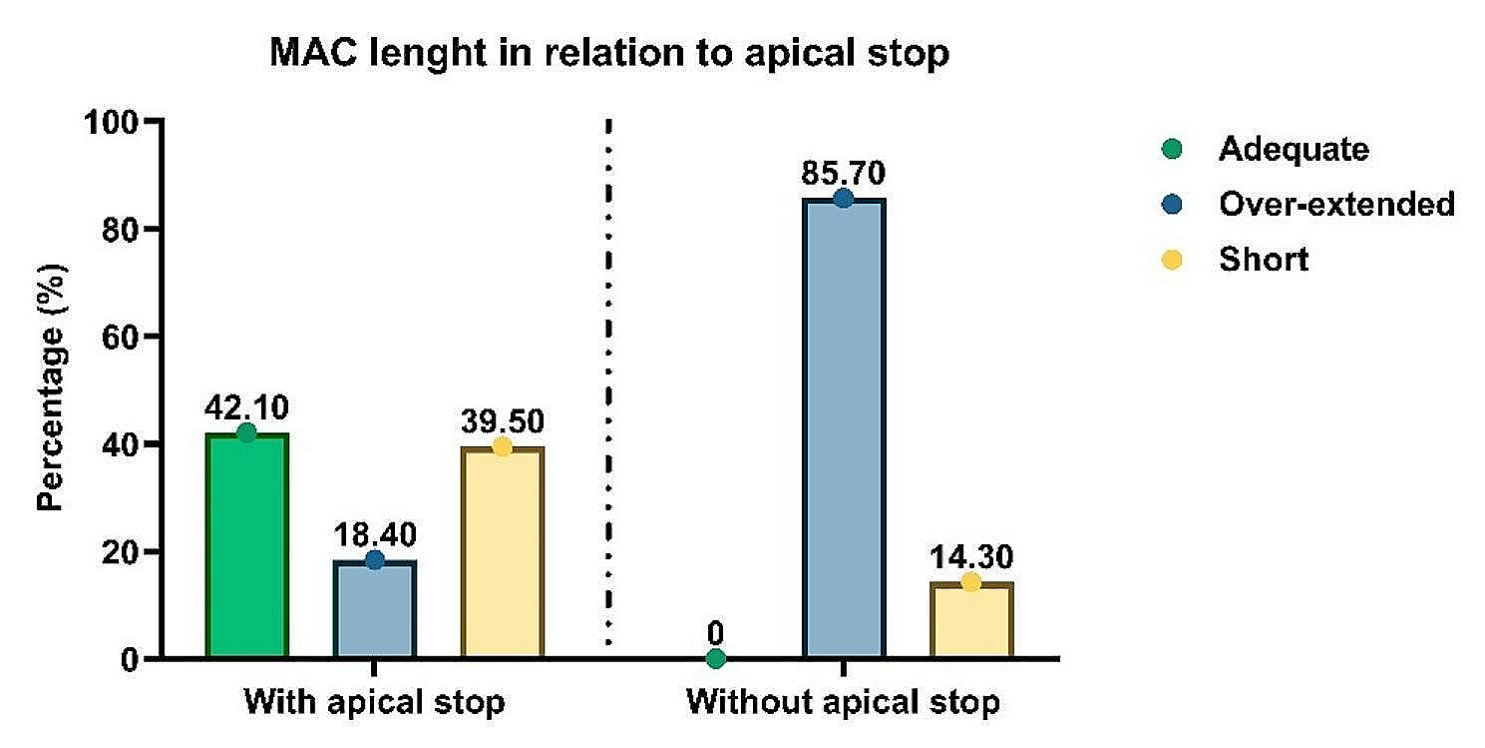
Percentages of adequate, over-extended, and short master apical cone (MAC) length with and without an apical stop. A statistically significant correlation was found between MAC length and apical stop presence (*p* = 0.001)

**Fig. 4:**
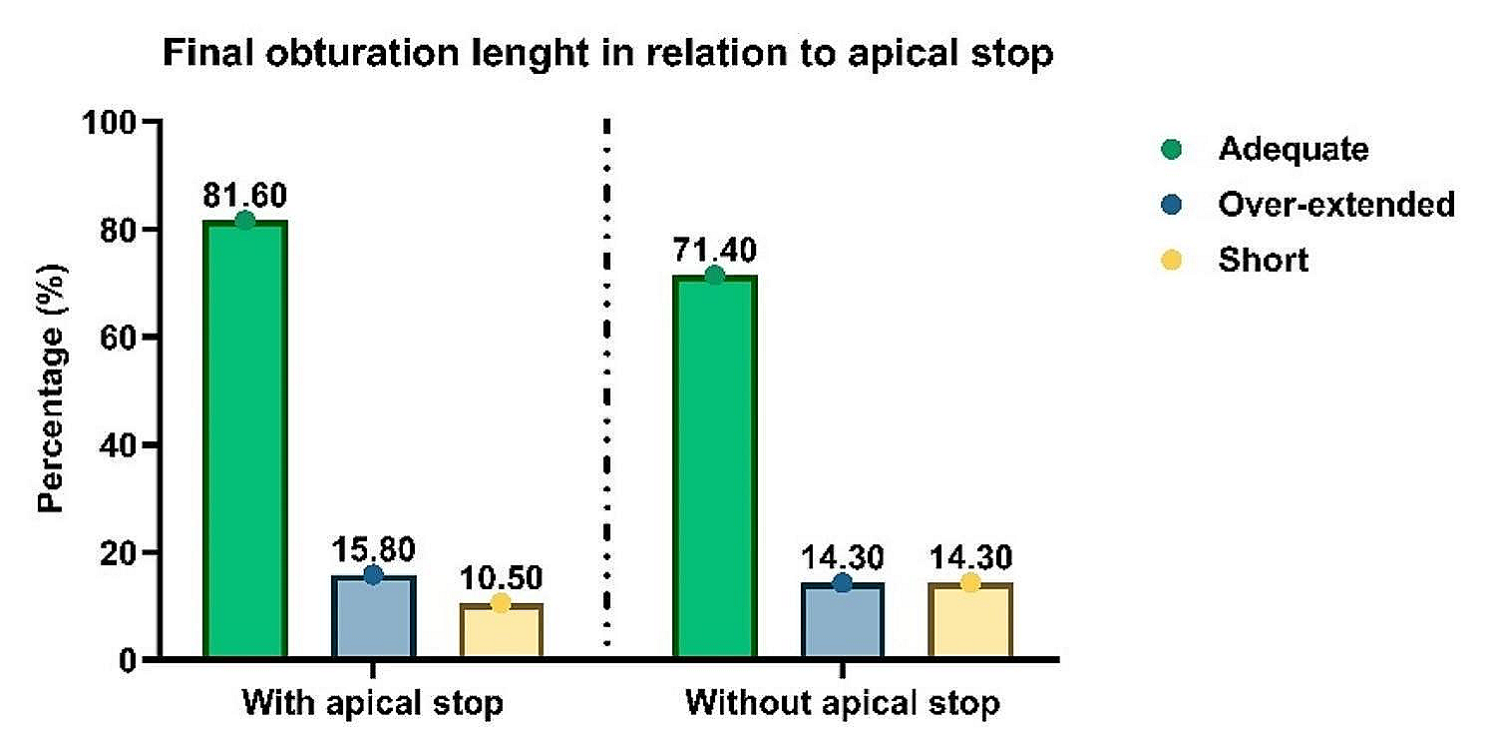
Percentages of adequate, over-extended, and short final obturation with and without an apical stop. There was no statistically significant correlation between apical stop and final obturation length (*p* = 0.809)

Only 55.6% (*n* = 25) of the students felt tug-back; however, most participants (84.4%, *n* = 38) felt an apical stop. During the MAC radiograph stage, 35.6% (*n* = 16) of the students used an adequate cone length. The master GP cone was found to be of adequate length in the first MAC radiograph for 16 students (35.6%), over-extended for 13 students (28.9%), and short for 16 students (35.6%). No significant correlation was found between the presence of tug-back and master GP cone length on MAC radiograph or between the presence of tug-back and obturation length on the final radiograph (*p* = 0.408 and *p* = 0.149, respectively). On the other hand, a significant correlation was found between the presence of an apical stop and the length of the master GP cone on MAC radiograph (*p* = 0.001). However, there was no correlation between obduration length and presence of an apical stop on the final radiograph (*p* = 0.809; Figs. 3 and 4).

In the 30 cases with improper cone fitting, the main problem was improper canal shaping for 25 students, (83.3%), and only 5 students (16.7%) did not choose the correct cone size. One of those students changed the cone because it fit loosely inside the canal even though the length on the MAC radiograph was adequate. Fifteen students who shaped root canals inadequately were instructed to re-instrument them to ensure that the root canals reached their full length.

The final obturation length was adequate in 80% of the cases (*n* = 36). Most students (57.8%, *n* = 26) were assisted by their instructors. Statistically, there was no significant correlation between help from instructors and final obturation length (*p* = 0.110). Eleven (55%) fourth-year students took only one MAC radiograph, whereas eleven (44%) fifth-year students took two radiographs to confirm the MAC length. However, there was no significant difference between the levels (*p* = 0.39). The intraclass correlation between the four observers was 0.774, indicating good agreement [21]. The value of Fliess’ kappa ranged from 0.83 to 1 for intra-observer reliability, indicating almost perfect agreement among all observers, and the value was 0.755 for the inter-observer reliability test, indicating substantial agreement [22]. The results of Fliess’ kappa are summarised in Table 4.

**Table 4:** Summary of results of Fliess’ kappa for intra- and inter-observer reliability

Observer	A	B	C	D
A	0.83*	1*	0.7^+^	0.85*
B	1*	1*	0.7^+^	0.85*
C	0.7^+^	0.7^+^	1*	0.5^^^
D	0.85*	0.85*	0.5	0.85*
Overall	0.755 ^+^			

A, B, C and D: The four observers included in the study

^^^ Moderate agreement

^+^ Substantial agreement

* Almost perfect agreement

## Discussion

The primary objective of obturation is to seal the cleaned and shaped root canal space, and cone selection is one of the critical steps required to achieve this goal [16, 23]. Procedural errors can occur during obturation among undergraduate dental students due to their insufficient knowledge and experience [17, 24]. In the first phase of this study, we aimed to evaluate the students’ knowledge of master GP cone selection through an online survey sent to all fourth- and fifth-year students. In the second phase of the study, we observed actual performances of master GP cone fitting by these students during clinical endodontic courses at the College of Dentistry, King Saud University. Throughout this study, we implemented the same evaluation parameters adopted in our pre-clinical and clinical courses. In those courses, students’ knowledge was assessed through the written exams, while the performance of cone selection and fitting was assessed in a clinical setting, using both self-evaluation by the students and evaluation by their supervisors.

This investigation allowed us to detect problems that led to obturation-related mishaps. To our knowledge, no previous studies have explored this aspect of dental students’ knowledge and performance.

### Phase I: knowledge survey

In this study, most students demonstrated a good knowledge of GP cone selection and were able to define tug-back and apical stops. This can be attributed to the fact that all participants passed the pre-clinical endodontic course, which consisted of a series of lectures and laboratory exercises, on, among other things, obturation and GP cone selection techniques. However, some students had insufficient knowledge and expressed confusion regarding the assessment of apical stops and tug-back. Weaker students might have difficulty learning new concepts and should be given more attention during teaching activities [25].

Regarding the question about root canal flaring assessment techniques, a significant difference was found between the responses of male and female participants. According to Allison et al., root canals should be shaped in a way that allows the insertion of a spreader with a fitted cone 1–2 mm from the working length [26]. At our institute, male and female students at the undergraduate level occupy different university campuses. Thus, the difference in responses could be due to different terminologies being taught at the two campuses. Alternatively, perhaps this question was clear only to the female students because they were used to communicating with the investigators. Whatever the case, it is ideal to standardise teaching across different campuses.

All students were able to identify the different methods of managing an overextended GP cone. The ideal management is re-instrumenting the canal and creating an apical seat, an incomplete barrier at the apical end of the root canal preparation [20]. However, in some cases, it is difficult to prepare an apical seat, and GP cone adjustment is the only option. Cutting the tip of the cone, and using a larger cone are valid approaches that facilitate achieving the apical stop at the determined WL [27–29].

### Phase II: clinical skills observation

During our clinical observation, the length of each master GP cone and final obturation were assessed radiographically. Radiographic interpretation has always been known as a subjective form of assessment. Goldman et al. reported 47% inter-observer agreement for radiographic interpretation [30]. In our study, the overall inter-observer Fliess’ kappa value was 0.755, indicating substantial agreement among the observers [21].

One of the most important steps of a successful root canal treatment is establishing an accurate working length based on a reliable reference point on the tooth structure [31]. Improper length control can result in over-instrumentation or apical perforation and subsequent overfilling. This can increase postoperative pain and delay or impede healing [13, 32]. We observed that most students used a tooth structure as a reference point. It is recommended to level off cusp tips and incisal edges that serve as reference points with fissure carbide and diamond burs.

While observing the procedures, we found that students used different methods, either bending or making an indentation to label the WL on the master GP cone before taking the MAC radiograph. A given student’s preferred technique might be influenced by their instructors or what they learn from their classmates. Labelling the cone with an indentation is recommended by scholars cited in our curriculum. According to our best knowledge, however, bending the GP cone was not mentioned in any previous publications, and the effect of different labelling methods on treatment outcomes is unknown [3].

Before conducting this observation, we noticed that when students bent the GP cone at the reference point, the length of the master cone would appear optimal on the MAC radiograph, even without an apical stop. Afterward, the student would proceed with obturation, in which the cone would be extruded through the lateral condensation technique, resulting in overfill. Although our current clinical observations failed to find any significant impact of the GP labelling technique on the length of the master GP cone or the final obturation, we advise marking the WL with an indentation or by grasping the cone with a tweezer, as previously described [1].

In the current investigation, the initial apical stop was found to have a significant impact on GP cone length on the MAC radiographs. The student made adjustments to the cone or re-instrumented the canals as advised by the instructor, whenever the cone was either short or over-extended. Thus, the initial apical stop had no impact on the final obturation length. This finding doesn’t reduce the significance of the apical stop. However, it demonstrates that even without the presence of the apical stop initially, you can still achieve an appropriate length by effectively managing the selection of the cone based on the MAC radiograph. This emphasised to the undergraduate students the importance of taking MAC radiographs. However, the tug-back sensation had no effect on length. In contrast, Saatchi et al. suggested that tug-back is important and can enhance the apical seal [33].

Our results indicated that the most common cause of ill-fitting cones was improper shaping of the canal. These findings emphasise the importance of cleaning and shaping [34]. Therefore, we recommend focusing teaching at the undergraduate level more on cleaning and shaping principles, including the management and prevention of procedural accidents.

At our institute, students are trained to perform the manual step-back technique in the first semester of pre-clinical endodontic training, and the NiTi rotary systems (ProFile system) are introduced in the second semester [35, 36]. Later, in the clinical endodontic courses, students have the choice to use either manual or rotary instrumentation. Most students in our sample preferred the use of rotary tools over hand instrumentation, perhaps because endodontic rotary systems enable the student to complete the procedure faster and with fewer errors [19, 37, 38]. However, hand instrumentation techniques should be practiced in addition to rotary skills to maintain and develop the relevant tactile sensations [39].

We expected that fifth-year students would be more competent than fourth-year students during the GP cone selection [40]. However, the number of fifth-year students who took only one MAC radiograph (56%) was comparable to that of fourth-year students (55%). These unexpected results may be attributed to the pandemic that started in 2020, causing an abrupt interruption in the education of fifth-year students at the preclinical stage. Furthermore, fourth-year students get the benefits of extra careful supervision in comparison to fifth-year students. The majority of the observed students (57.8%) depended on the instructor’s guidance, indicating that most students are unable to independently perform cone selection. These findings emphasise the important role of pre-clinical training and close monitoring for undergraduate students. Instructors should take on the responsibility of guiding and offering verbal feedback with clinical demonstration to students, rather than confining their role solely to evaluation. This approach ensures a comprehensive and efficient educational process and experience.

A prior endodontic clinical observation by Alghamdi et al. found that obturation-related mishaps accounted for the majority of errors made by undergraduate dental students. They reported 19.6% of the cases to be overfilled and 68.1% of the cases to have short obturations [6]. Moreover, a radiographic assessment by Matoug-Elwerfelli et al. reported around 45% obturation-related mishaps [41]. The lower incidence of obturation-related errors observed in our current study may have been caused by the smaller sample size and the close supervision during the master GP cone fitting and selection.

### Limitations of the study

The response rate for the online survey was 62%. This low rate might be due to the survey fatigue phenomenon described in previous reports [42, 43]. We tried to reduce survey fatigue by reducing the number of questions, omitting open-ended queries, and providing an estimate of how long it would take to complete the survey. In addition, the Hawthorne effect should be considered a limitation on the clinical observations. The Hawthorne effect is a change in observed individuals’ behavior caused by the knowledge or feeling of being observed during a study, which can lead to a false-positive bias and overoptimistic results [44, 45] We tried to minimise the Hawthorne effect in two ways. First, consent forms were signed two weeks before the clinical observations. Second, the observers were the same clinical instructors the students usually had during their clinical sessions, so the students did not know when they were being observed for our study.

Moreover, the sample size of the clinical observation was relatively small, and only female students were included, as male students are trained on a different campus. Thus, the results of the current study cannot be generalised to male students, and future studies with larger samples are recommended.

## Conclusion

Within the limitations of the study, it can be concluded that most of the undergraduate dental students had the basic knowledge required to solve different problems related to the selection and fitting of master GP cones. However, in the clinical context, more than half of the students required the assistance of their instructor to adjust and fit the master GP cone. The presence or absence of an apical stop had the most significant effect on the length of the fitted master GP cone based on the radiograph results. The major cause of ill-fitting master cones was improper shaping of the canal. Therefore, endodontic teaching should focus more on the skills of canal cleaning and shaping.

## Data Availability

All generated data used and/or analysed during the current study were included within the manuscript.

## Abbreviations

RCT: Root canal treatment
GP: Gutta-percha
WL: Working length
MAC radiograph: Master apical cone radiograph
